# 68-channel neural signal processing system-on-chip with integrated feature extraction, compression, and hardware accelerators for neuroprosthetics in 22 nm FDSOI

**DOI:** 10.3389/fnins.2024.1432750

**Published:** 2024-10-23

**Authors:** Liyuan Guo, Annika Weiße, Seyed Mohammad Ali Zeinolabedin, Franz Marcus Schüffny, Marco Stolba, Qier Ma, Zhuo Wang, Stefan Scholze, Andreas Dixius, Marc Berthel, Johannes Partzsch, Dennis Walter, Georg Ellguth, Sebastian Höppner, Richard George, Christian Mayr

**Affiliations:** ^1^Faculty of Electrical and Computer Engineering, School of Engineering Sciences, Dresden University of Technology, Dresden, Germany; ^2^Department of Electrical and Computer Engineering, College of Engineering, University of Utah, Salt Lake City, UT, United States

**Keywords:** biomedical signal processing, neural recording system, digital integrated circuits, neural signal compression, implantable devices, biomedical electronics, spike sorting

## Abstract

**Introduction:**

Multi-channel electrophysiology systems for recording of neuronal activity face significant data throughput limitations, hampering real-time, data-informed experiments. These limitations impact both experimental neurobiology research and next-generation neuroprosthetics.

**Methods:**

We present a novel solution that leverages the high integration density of 22nm fully-depleted silicon-on-insulator technology to address these challenges. The proposed highly integrated programmable System-on-Chip (SoC) comprises 68-channel 0.41 μW/Ch recording frontends, spike detectors, 16-channel 0.87–4.39 μW/Ch action potentials and 8-channel 0.32 μW/Ch local field potential codecs, as well as a multiply-accumulate-assisted power-efficient processor operating at 25 MHz (5.19 μW/MHz). The system supports on-chip training processes for compression, training, and inference for neural spike sorting. The spike sorting achieves an average accuracy of 91.48 or 94.12% depending on the utilized features. The proposed programmable SoC is optimized for reduced area (9 mm^2^) and power. On-chip processing and compression capabilities free up the data bottlenecks in data transmission (up to 91% space saving ratio), and moreover enable a fully autonomous yet flexible processor-driven operation.

**Discussion:**

Combined, these design considerations overcome data-bottlenecks by allowing on-chip feature extraction and subsequent compression.

## 1 Introduction

Recent advances in the analysis of neuronal microcircuits and the design of neural prosthetic devices, demand the analysis of neural activities recorded with high spatial and temporal resolution from 100's or even 1,000's of channels (George et al., 2020). However, transmitting such a substantial volume of neural signals off-chip proves to be highly power-consuming, constrained by bandwidth limitations, and poses challenges in terms of storage capacity, rendering some significant applications of extracellular signals such as brain-machine interfaces and neural prosthetics challenging.

Conventional neural signal acquisition systems typically consist of analog frontends (AFEs) responsible for amplifying, filtering, and digitizing raw data. Here, an increase in electrodes causes a proportional rise in throughput, memory, and power requirements. Analysis from Zeinolabedin et al. (2022) suggests that a conventional 1000-channel recording system requires a power budget of ~250 mW for off-chip transmission of raw neural signals. Yet, to ensure thermal biocompatibility of an implantable system, power consumption must remain below 35 mW, as demonstrated by the 3D Utah electrode array (Kim et al., 2008). Furthermore, bandwidth analysis reveals the limitations of conventional acquisition approaches. For instance, assuming a sampling frequency of 20 kHz and each sample comprising 9 bits, a recording system with 1,000 channels necessitates a bandwidth of at least 180 Megabits per Second (Mbps). Consequently, integrating on-chip digital processing engines becomes imperative (Shaeri et al., 2024).

**Figure 1:**
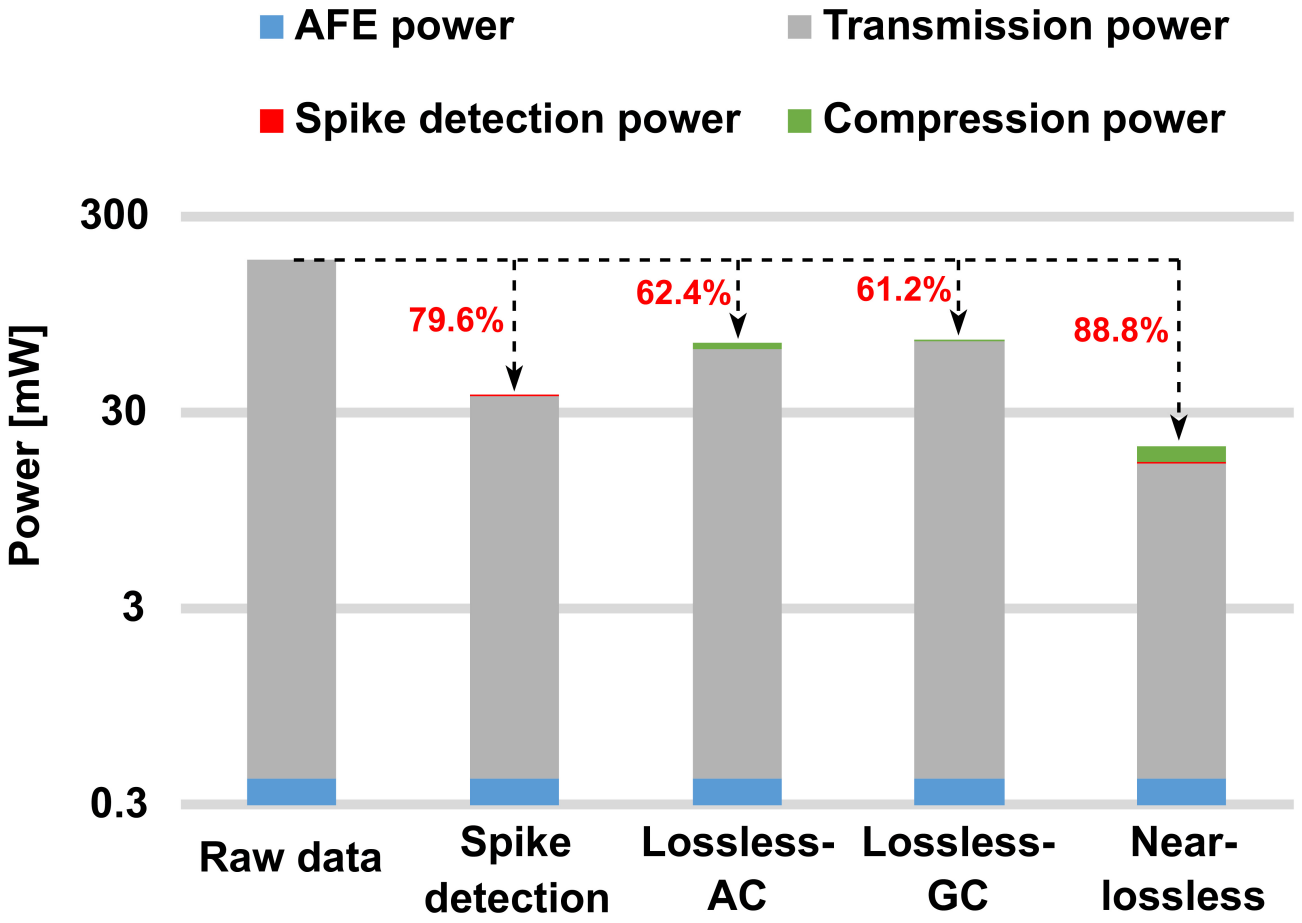
Power analysis of a 1000-channel neural recording system. For the proposed programmable SoC: P(AFE) = 0.41 μW/Ch, P(Lossless-AC) = 4.39 μW/Ch, P(Lossless-GC) = 0.87 μW/Ch, P(Near-lossless) = 3.24 μW/Ch, firing rate = 60 spikes/sec, transmission energy = 1 nJ/bit, sampling frequency = 20 kHz, and the data bit-width = 9 bits. AC, arithmetic coding; GC, Golomb coding.

With a focus on alleviating these limitations, a crucial subset of on-chip digital processing engines is dedicated to spike processing, specifically spike detection and spike sorting. If the average neuronal firing rate is ~60–100 spikes per second, with each spike having a window size of 64 samples under a sampling rate of 20 kHz, spike detection would theoretically reduce the data rate to about 36.2–59.6 Mbps for a 1000-channel recording system. This includes the transmission of inter-spike intervals and channel indexes to ensure data reconstruction. This reduction in data rate translates to a potential power reduction of about 66.6–79.6% compared to conventional recording systems, as shown in Figure 1. Spike sorting has the potential to further reduce data rate and power consumption by transmitting only the spike index instead of the entire spike. Reference Chae et al. (2009) reports a spike detection and feature extraction (FE) for spike sorting, albeit limited to processing a single channel at a time. In Karkare et al. (2013), a 16-channel System-on-Chip (SoC) performing training and inference on-chip is introduced, specifically designed for the *OSort* online training algorithm described in Rutishauser et al. (2006). However, it suffers from a large memory requirement and a limited number of channels due to its power consumption. Reference Zeinolabedin et al. (2022) performs multi-channel on-chip spike detection, feature extraction, and training and inference for spike sorting with low power consumption. Nonetheless, its capability is restricted to performing Euclidean distance metrics, which shows limitations for some datasets (Guo et al., 2022).

Figure 1 reveals another critical subset of on-chip digital processing engines, that is compression. Unlike spike sorting, compression offers enhanced potential for off-chip signal reconstruction, particularly with lossless compression methods. This capability provides significant advantages, especially in explorative neuroscientific applications, where a visual inspection of the raw signal is required. Additionally, compression finds broader utility across various contexts. For instance, in the context of local field potentials (LFPs) and multi-unit activity (MUA), spike sorting proves inadequate. This point will be further elucidated in the following section, along with the characterization of LFPs. According to Guo et al. (2023), the compression of action potentials (APs) is categorized into three groups: lossless compression, near-lossless compression, and lossy compression. Reference Bonfanti et al. (2010) introduces a compression engine (CE) employing a hard thresholding spike detection and Manchester code, achieving a near-lossless space saving ratio (SSR) of 87.8%. Lossy compression is often performed in the frequency domain. Reference Hosseini-Nejad et al. (2014) utilizes the Walsh-Hadamard transform, followed by a threshold operation to filter out certain coefficients, achieving an SSR of nearly 90% with a firing rate of ~55–60 spikes/sec. Similarly, Shaeri et al. (2011) and Kamboh et al. (2009) employ discrete wavelet transforms. Reference Thies and Alimohammad (2019) converts spikes into a feature space to achieve a lower data rate; however, it fails to encode important interspike intervals necessary to recover precise spike timings. These methods achieve high SSR at the cost of introducing compression artifacts. In Guo et al. (2023), a combined lossless and near-lossless compression engine is proposed; however, its scalability is limited by a power consumption of ~17 μW/Ch. Regarding LFPs, Cuevas-López et al. (2022), Wang et al. (2024), Valencia et al. (2024), Khazaei et al. (2020), Nurse et al. (2016), and Schmale et al. (2013) present various compression algorithms along with temporal and spatial decorrelation methods, yet they suffer from challenges such as high hardware complexity, imperfect reconstruction, or insufficient SSR.

Another highlight of the proposed programmable SoC is that it integrates an ultra-low power reduced instruction set computer (RISC-V). In settings where electrode movement and tissue reactions are commonplace, as observed in contemporary high-density multi electrode arrays such as Ballini et al. (2014), Han et al. (2013), Lopez et al. (2016), and Schüffny et al. (2022), the significance of on-chip programmability and re-training, cannot be overstated. Its role is pivotal in facilitating real-time adaptation, enabling dynamic adjustments to compression engines and spike-sorting classifiers, thereby ensuring sustained high performance in SSR and classification accuracy. To meet these demands, the ultra-low power RISC-V is complemented by hardware accelerators. Despite the critical nature of this requirement, contemporary state-of-the-art works integrating a core are rare. Furthermore, there are notable designs such as Karkare et al. (2013), Karkare et al. (2011), and Chen et al. (2023) that emphasize various on-chip data processing aspects but do not incorporate analog frontends, hindering their applicability in implantation scenarios.

Following these design considerations, the programmable System-on-Chip presented, comprises:

- A Digital Signal Processing Wrapper (DSPW) containing a Central Bio-Signal Processing Unit (CBPU), a compression engine consisting of Intra- and Cross-Channel Compression Engines (16 × ICE and CCE), a spike raster (SR), an Adaptive Threshold Estimator (ATE), and a finite impulse response (FIR) filter.
- An on-chip ultra-low power RISC-V, handling primarily the training process of CCE, the training and inference process of spike sorting, and other processor-driven on-chip operations.
- A configurable general purpose multiply-accumulate (MAC) unit which accelerates neural processing such as spike sorting. For general use cases, this module is supplied by static random access memory (SRAM). Dimensions and features of the MAC unit can be adapted via the control register block of the Central Bio-Signal Processing Unit control registers.
- A 68-channel delta-sigma 0.4 V 9-bit analog-to-digital converter (ADC) with corresponding 0.55 V Analog-Digital Wrappers (ADW). The analog frontend is capable of seamless switching between high-bandwidth and low-bandwidth modes.

The programmable SoC is implemented using 22 nm fully-depleted silicon-on-insulator (FDSOI) technology. The acquisition side (ADC and ADW) achieves a power consumption of 0.41 μW per channel. Additionally, the Intra-Channel Compression Engine achieves an SSR of ~91% for action potentials in the near-lossless mode and an SSR of 64% for LFPs. The ultra-low power RISC-V consumes a dynamic power of 5.19 μW/MHz. The MAC unit decreases the power consumption of the software-based on-chip spike sorting by ~23.8%, equivalent to 1.09 μJ/spike, achieving an average accuracy of 91.48 or 94.12% based on utilized features over datasets published in Quiroga et al. (2004).

The subsequent sections of this paper are structured as follows. Section 2 provides a concise overview of the fundamentals of neural signal processing approaches, detailing the proposed architecture and its constituent components. Section 3 presents the results of the measurements. Section 4 delves into a comparative analysis, followed by concluding remarks.

## 2 Materials and methods

### 2.1 Fundamentals of neural signal processing approaches

This subsection is dedicated to characterizing the extracellular signal and discussing prevalent neural signal processing methodologies. This aims to underscore the advanced integration capabilities of the proposed programmable SoC, demonstrating its versatility as a platform for capturing extracellular neural signals.

Brain activities can be categorized into four types based on recording positions: electroencephalography (EEG), electrocorticography (ECoG), extracellular signals, and intracellular signals. Due to their spatial resolution and acquisition complexity, extracellular signals have garnered significant attention (Dipalo et al., 2017). This work primarily concentrates on recording and processing extracellular neural signals. Typically, extracellular signals comprise two main components:

- Action potentials: APs represent rapid rises and falls of the membrane potential of neurons, which have typical a frequency of 100 Hz–10 kHz (Hodgkin and Huxley, 1990; Zeinolabedin et al., 2022). Also referred to as spikes, action potentials serve as fundamental electrical signals crucial for communication within the nervous system. Their dynamics are integral to deciphering the complex mechanisms governing brain function and behavior.
- Local field potentials: LFPs are derived from the lowpass filtering of raw extracellular signals recorded from neural tissue surrounding the electrode, typically within a diameter of ~1 mm (Buzsaki et al., 2012; Rey et al., 2015). These LFPs are pivotal in various applications, including the detection of conditions such as Alzheimer's, Parkinson's disease and epileptic seizures (Rowland et al., 2015; Wu et al., 2013; Zeng et al., 2023; Hou et al., 2024).

In addition to action potentials and LFPs, extracellular signals include other components such as MUA. While MUA provides valuable information for broader measures like average firing frequency and time-to-first-spike, as demonstrated in studies such as Velliste et al. (2008) and Flint et al. (2013), it is generally considered background noise in spike-sorting scenarios (Buzsaki et al., 2012). Since the methods involved in the analysis of such signal features are highly application-dependent, no dedicated hardware accelerator was considered for this task.

The conventional approach to processing action potentials acquired through extracellular recording, is to use bandpass-filters, and subsequent spike detection and spike sorting. Compared to spike detection and transmitting entire spikes, spike sorting has the potential to conserve more bandwidth and reduce power consumption. Nevertheless, spike sorting methods encounter a significant limitation: the inability to reconstruct the transmitted signal off-chip. This limitation poses challenges for applications such as neuroscientific research and clinical practice (Guo et al., 2023) when compared to traditional neural signal acquisition approaches. Moreover, transmitting raw neural signals offers the potential for algorithmic development in spike detection and sorting. Thus, on-chip compression of neural signals becomes crucial as well, particularly focusing on the lossless or near-lossless compression (Guo et al., 2023; Ma et al., 2021).

The typical processing steps for LFPs encompass several key stages, including filtering, feature extraction, the computation of biomarkers, and more (Jackson and Hall, 2017; Navas-Olive et al., 2022; Summerson et al., 2015). Additionally, similar to action potentials, transmitting raw LFPs off-chip is crucial for off-chip data analysis and algorithm development, underscoring the significance of LFP compression as a meaningful processing step. In contrast to action potentials, LFPs exhibit clear spatial correlation, necessitating a distinct approach to compression (Cuevas-López et al., 2022; Wang et al., 2024; Valencia et al., 2024; Khazaei et al., 2020; Nurse et al., 2016; Schmale et al., 2013).

### 2.2 Proposed system architecture

**Figure 2:**
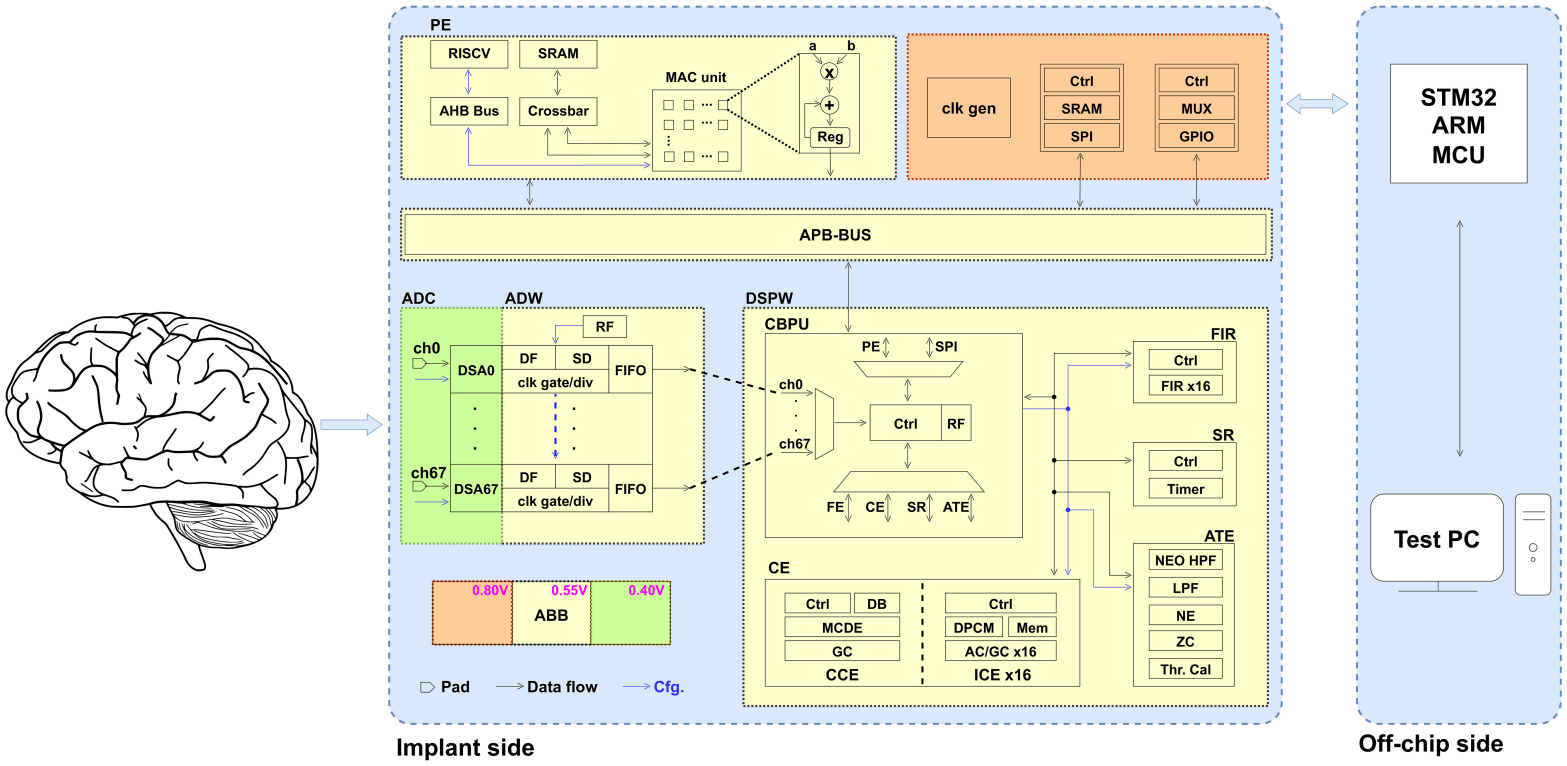
System architecture of proposed programmable SoC. The SoC consists of three power domains and the following main components: acquisition analog frontends, digital processing wrapper, MAC-assisted PE, and periphery. AHB, Advanced High-Performance Bus; DB, data buffer; DPCM, differential pulse-code modulation; DSA, delta-sigma ADC; HPF, high-pass filter; LPF, low-pass filter; MCDE, Multi-Channel Decorrelation Engine; MCU, microcontroller unit; MUX, multiplexer; NE, Noise Estimator; RF, register file; ZC, Zero Crossing.

Figure 2 illustrates the system architecture of the proposed programmable SoC, which is composed of three distinct power domains. The digital processing units, encompassing Analog-Digital Wrappers, Digital Signal Processing Wrapper, MAC-assisted processing element (PE), and the Advanced Peripheral Bus (APB), are powered at a domain of 0.55 V, facilitated by adaptive body-biasing (ABB) technology, the implementation methodology of which is detailed in Höppner et al. (2020). The delta-sigma modulators of the analog frontend are robustly powered at 0.4 V, through adaptive back-gate voltage tuning (Schüffny et al., 2022) to compensate process, voltage, and temperature (PVT) variation. The third power domain operates at 0.8 V and supplies components such as serial peripheral interface (SPI) and general purpose input/output (GPIO).

The extracellular neural signal undergoes initial recording through the 68-channel frontends. The configurable ADCs, coupled with digital filters (DF) and spike detectors (SD), facilitate an effective trade-off between high resolution and ultra-low power consumption. For non-spiking parts, the default low-bandwidth mode is employed. While spikes are detected, the high-bandwidth mode is activated. The two-stage spike detection, encompassing (1) adaptive thresholding and (2) nonlinear energy operator (NEO), ensures accuracy by testing the false positives (Schüffny et al., 2023b).

Subsequently, the neural signals are temporarily stored in 68 small first-in-first-out (FIFO) buffers located in Analog-Digital Wrappers, under the control of the Central Bio-Signal Processing Unit. This arrangement aims to maintain a specific order of inputs from all analog frontends, thereby ensuring the synchronization of data for potential applications such as spatial decorrelation.

The neural signal is then transmitted to the Digital Signal Processing Wrapper for the subsequent on-chip processing. The Central Bio-Signal Processing Unit supports nine distinct commands (C1–C9), each of which is highly configurable to meet the requirements in various application scenarios. The commands are described briefly as follows:

- C1: One of the primary commands involves recording neural signals from analog frontends and then transmitting the collected data to PE SRAM, facilitating on-chip data processing capabilities, such as data analysis, and training for Cross-Channel Compression Engine and spike sorting. Configurable parameters including channel selection, the frequency of information packets, the address information, and others, are managed through a register file.
- C2: In addition to on-chip data processing, another crucial application involves transmitting neural signals externally for off-chip data processing. Configurable parameters include channel selection, the frequency of information packets, the transmitting mode (stream/batch), and others.
- C3: In comparison to transmitting raw neural signals, the transmission of compressed data significantly reduces power consumption and conserves bandwidth. Command C3 is primarily employed for Intra-Channel Compression Engine and compression related to AP. Configurable parameters encompass channel selection, CE algorithm selection, compression mode (lossless/near-lossless), transmitting mode (stream/batch), and others.
- C4: Command C4 is specifically designed for Cross-Channel Compression Engine, primarily utilized for the compression of LFP. Configurable parameters include channel selection, feedback to the power management unit, transmitting mode (stream/batch), and others.
- C5, C6: The commands C5 and C6 are both allocated for utilization within the FIR module. Distinctions arise in their operational outputs: C5 entails transmission of the filtered signals via SPI, whereas C6 involves storage of the signals in the PE SRAM for subsequent on-chip analysis. Additionally, the module serves to filter out action potentials from raw data, thereby enabling applications such as seizure detection. Configurable parameters encompass channel selection, weights, among others.
- C7: Command C7 is specifically allocated to the spike raster module. Through the integration with the analog frontend and the two-stage spike detection module, the spike raster and CBPU control logic subsystems collaborate to produce timing-accurate spike raster plots. These plots serve a pivotal role in various applications, notably in the reconstruction and analysis of neural circuits.
- C8, C9: Commands C8 and C9 are purposefully designed for the Adaptive Threshold Estimator module, serving as the second stage of spike detection. Its primary function revolves around gathering the dynamic thresholds of channels and subsequently transmitting the obtained results off-chip for analysis (C8). Additionally, it facilitates the updating of individual first-stage spike detection parameters (C9).

**Figure 3:**
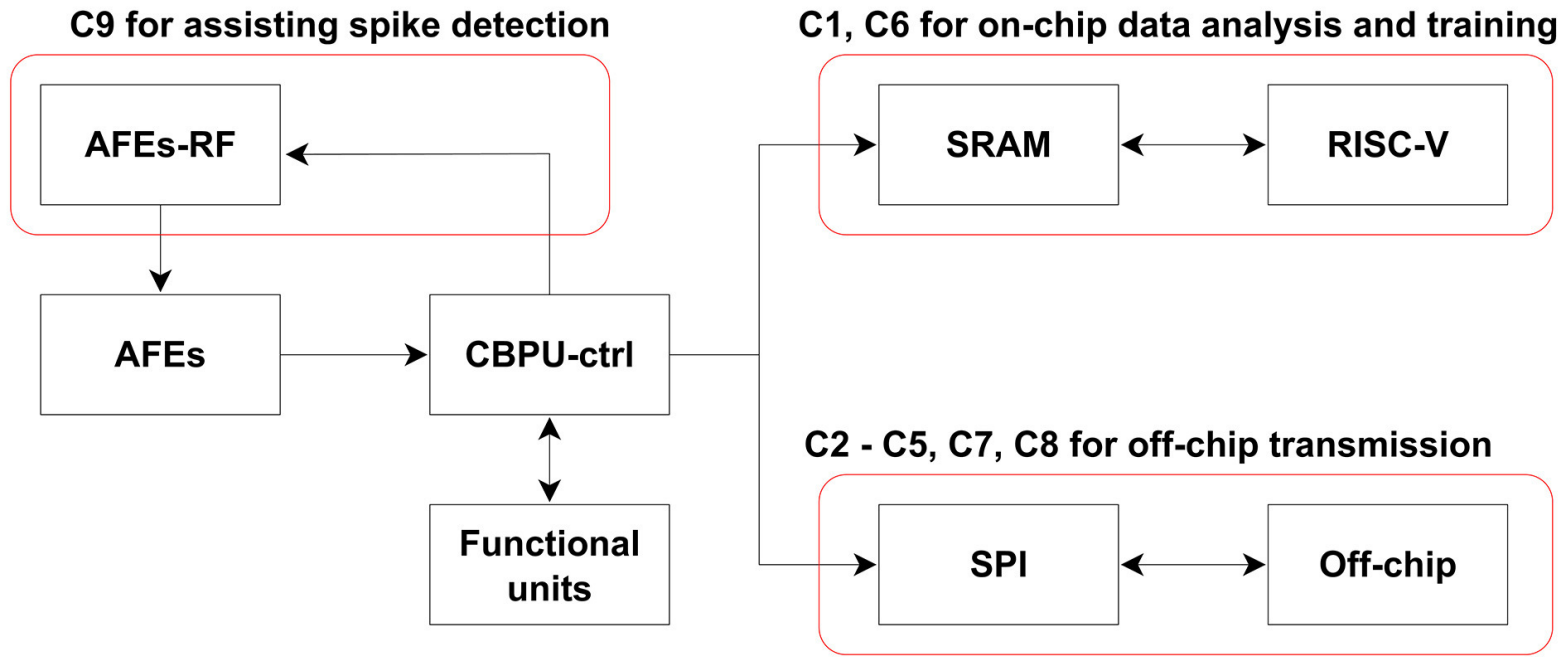
Data flow and active blocks depicting various commands. In addition to the labeled block in the diagram, each command involves the Central Bio-Signal Processing Unit control logic and corresponding functional units. For commands C1 and C6, the process comprises a recording phase followed by an analysis/training phase. During the recording phase, the RISC-V remains in sleep mode to conserve power.

The data flow and corresponding active blocks are illustrated in Figure 3. The commands can be categorized into three types based on their applications: off-chip data transmission, on-chip data analysis and training, and assistance for spike detection.

To verify the functionality of each functional block, a debug mode has also been implemented for commands C2, C3, C4, C5, C7, C8, and C9. In this mode, the Central Bio-Signal Processing Unit control logic retrieves data from the PE SRAM instead of the analog frontend, allowing users to pre-store data. The debug mode facilitates the debugging process by enabling users to compare the outputs of individual functional modules with the software results obtained off-chip, thereby verifying system performance and aiding in the development of recording protocols. Activation of the debug mode is controlled by a specific bit in the commands.

### 2.3 Digital signal processing wrapper

**Figure 4:**
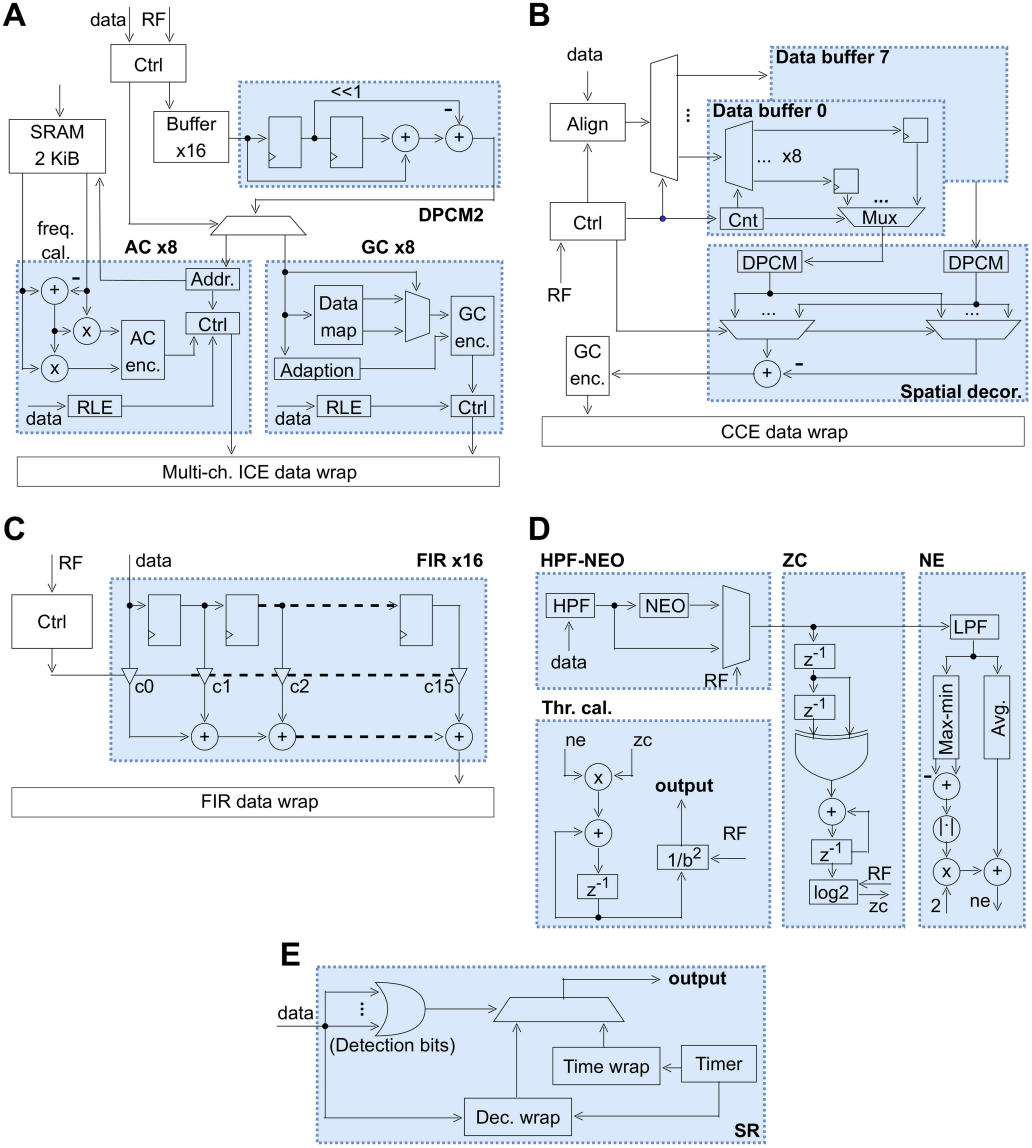
Block diagrams of main components in the DSPW. **(A)** Block diagram of ICE. **(B)** Block diagram of CCE. **(C)** Block diagram of the FIR filter. **(D)** Block diagram of ATE. **(E)** Block diagram of SR. DPCM2, 2nd-order differential pulse-code modulation; KiB, Kibibyte; RLE, run-length encoding.

As illustrated in Figure 2, the proposed programmable SoC incorporates a versatile Digital Signal Processing Wrapper. This subsection provides a comprehensive explanation of the primary functional components comprising the Digital Signal Processing Wrapper, the block diagrams of which are shown in Figure 4. These components fulfill crucial roles in facilitating diverse on-chip extracellular signal processing tasks.

#### 2.3.1 Intra-Channel Compression Engine for action potential compression

As highlighted in Section 2.2, the Central Bio-Signal Processing Unit control logic initially gathers data from either analog frontends in normal mode or the PE SRAM in debug mode. Subsequently, it forwards the data from specific user-selected channels to the Intra-Channel Compression Engine module for executing lossless or near-lossless compression. In prior research (Guo et al., 2023), an on-chip compression engine was developed, focusing on achieving high SSR, and utilizing adaptive arithmetic coding. Detailed power and area analysis reveals that ~75% of power consumption and 95% of area are occupied by the adaption module, opening a perspective for further system optimization.

Figure 4A illustrates the block diagram detailing the proposed Intra-Channel Compression Engine. This engine comprises 16 compression modules, with eight employing arithmetic coding (AC) and the remaining eight utilizing Golomb coding (GC). Notably, the system offers flexible configuration options, allowing each of the 68 analog frontends to be designated for compression using either AC or GC. Upon receiving data tagged with the designated channel index, it undergoes initial storage in a buffer with a capacity of 32. The underlying concept here is crucial for near-lossless compression, where upon detecting a spike, up to 32 preceding samples are considered part of the current spike, ensuring precise spike reconstruction. The subsequent step involves 2nd-order differential pulse-code modulation (DPCM2), a straightforward yet effective time-domain decorrelation technique. DPCM2 employs two previous samples to predict the current sample, while DPCM employs only one previous sample. They are defined as follows:


(1) $$\begin{array}{l} \begin{array}{c} DPCM=s\left(i\right)-s\left(i-1\right)\\ DPCM2=s\left(i\right)-2\cdot s\left(i-1\right)+s\left(i-2\right) \end{array} \end{array}$$

where *s* denotes the samples to be compressed. Compared to the DPCM method outlined in Guo et al. (2023), DPCM2 notably concentrates the raw data distribution, a critical aspect for enhancing the performance of entropy encoding algorithms such as AC and GC.

Initially, we delve into how the AC engine enhances area efficiency and reduces power consumption while incurring a minimal decrease in SSR. Unlike adaptive arithmetic coding, AC eliminates the need for an adaptive module. Instead, it employs a 2 Kibibyte (KiB) SRAM to store the distribution of all symbols, shared among eight AC engines. This distribution is established through training. Experimental findings indicate that multiple channels can share similar distributions, conserving memory space while ensuring each channel maintains a high SSR. This approach embodies a form of semi-adaptive compression engine.

In contrast to AC, the GC engine exhibits a slightly lower SSR; however, it compensates with unparalleled efficiency in both area and power consumption. Following decorrelation via DPCM2, the subsequent stage for GC involves an efficient data mapping process. The reasons are that (1) GC requires non-negative inputs, and (2) the smaller the inputs, the higher the SSR. The output from the data mapping module is then directed to the GC encoder. To optimize GC performance, an adaptation module is incorporated, primarily responsible for tracking the proportion of zero samples.

In the near-lossless mode, all samples between spikes are treated as zeros, and a run-length encoding (RLE) module is incorporated to quantify the length of these zero intervals. The RLE output is subsequently split into 2 or 3 components bitwise and transmitted to either arithmetic coding or Golomb coding modules as per the chosen encoding scheme. During decoding, data is grouped into sets of 66 or 67 samples. The initial 2 or 3 samples within each group are utilized to derive the RLE result, succeeded by a spike comprising 64 samples, denoting the end of the zero interval and the resumption of non-zero data.

In our Intra-Channel Compression Engine, we explored the implementation of both AC and GC to demonstrate their compatibility within a multi-channel action potentials compression engine. GC offers notable advantages in terms of area and power efficiency, rendering it particularly advantageous in scenarios with an extensive number of channels. However, in instances of highly noisy data, its SSR tends to be noticeably lower than that of AC. This aspect gains significance, especially in applications involving wireless transmission, as further discussed in Section 1.

#### 2.3.2 Cross-Channel Compression Engine for LFP compression

Compared to multi-channel action potentials, LFP exhibits a more pronounced correlation in the spatial dimension. This highlights the insufficiency of relying solely on temporal decorrelation to achieve a high SSR. Therefore, the primary objective of LFP compression is to efficiently decorrelate signals in both the temporal and spatial dimensions.

Figure 4B shows the block diagram of an 8-channel Cross-Channel Compression Engine designed for LFP compression. As explained, spatial decorrelation holds significant importance in LFP compression, necessitating precise alignment of data from various channels. This alignment process unfolds in two stages. Initially, Central Bio-Signal Processing Unit control logic undertakes the responsibility of aligning data from different analog frontends to ensure synchronization. Subsequently, within the Cross-Channel Compression Engine, the align module initiates the operation of data buffers upon the delivery of data from the channel with the smallest index among the selected eight channels. All eight data buffers used in Cross-Channel Compression Engine are tiny compared to the buffers used for Intra-Channel Compression Engine, since we do not need to care about buffering entire spikes.

Except for the root channel obtained during the training step, each channel has a single parent channel. This implies that the samples from the parent channel will be utilized for spatial decorrelation for the child channel. The basic formula of this process is defined as:


(2) $$\begin{array}{l} {ẽ}_{c}\left(n\right)=e_{c}\left(n\right)-\gamma \cdot e_{r}\left(n\right) \end{array}$$

where the suffix *c* denotes the child channel, the suffix *r* signifies the reference channel (parent channel). Additionally, *e*_*c*_ and *e*_*r*_ represent the temporally decorrelated signals of child and parent channels, respectively. The symbol γ denotes the spatial decorrelation factor, and ẽ_*c*_ is the temporally and spatially decorrelated signal, which serves as the data to be compressed for the corresponding child channel. To achieve the optimal decorrelation outcome, precise calculation of γ is essential. As per Kamamoto et al. (2008), there exists a positive correlation between the final coding length and energy, which in this context is defined as:


(3) $$\begin{array}{l} {Ẽ}_{c}=\overset{N}{\underset{n=1}{\sum }}{\left({ẽ}_{c}\left(n\right)\right)}^{2} \end{array}$$

where *N* denotes the length of the data segment used for computing γ. By taking the derivative of energy with respect to γ and solving for the point at which the derivative equals zero, we obtain:


(4) $$\begin{array}{l} \gamma =\frac{\sum_{n=n_{0}}^{N}e_{c}\left(n\right)\cdot e_{r}\left(n\right)}{\sum_{n=n_{0}}^{N}e_{r}\left(n\right)\cdot e_{r}\left(n\right)} \end{array}$$

where *n*_0_ indicates the starting point for γ calculation.

Equation 2 is commonly referred to as the one-tap mode. Alternatives include the three-taps mode or five-taps mode. The primary distinction lies in the number of values derived from the reference channel. These alternatives are also investigated in our experiments using datasets from Watson et al. (2016) and Vandecasteele et al. (2012). However, despite the evident increase in computational complexity with multi-taps mode, there is hardly any improvement observed in SSR.

The Cross-Channel Compression Engine requires a training process to establish the chain of channels and to calculate γ. As demonstrated in our experiments, we have verified that when the length of the data segment ranges from 1,000 to 2,000, the γ values of channels stabilize significantly. This indicates that on-chip training encounters no issues. Furthermore, a minimum spanning tree is utilized in the training process to ensure that the chain of channels does not form a closed-loop.

#### 2.3.3 FIR filter

For processing particular features of the neural signal e.g., LFPs, a discrete-time FIR filter is implemented based on the following formula:


(5) $$\begin{array}{l} \begin{array}{c} y\left[n\right]=c_{0}\cdot x\left[n\right]+c_{1}\cdot x\left[n-1\right]+\cdots +c_{N}\cdot x\left[n-N\right]\\ =\overset{N}{\underset{i=0}{\sum }}c_{i}\cdot x\left[n-i\right] \end{array} \end{array}$$

The implemented functionality can be utilized in concert with the PE, in order to efficiently implement the large group of commonly used algorithms that are based on convolutions. Among these, various transforms can be applied to the recorded discrete time series. For example, Discrete Wavelet Transforms, formulated as dyadic filter banks, can be employed to extract spectral features for event detection.

The Central Bio-Signal Processing Unit control logic receives data from the analog frontends and forwards the data to the filter module whenever new data arrives. The filter module is capable of dealing with data originating from up to 16 channels in parallel. It consists of a wrapper that instantiates 16 FIR taps, each of which has a filter length of 16. Each FIR tap is of order 15 and receives its data from the FIR data wrap module. The FIR data wrap maps the data from each of the 16 channels to one of the FIR taps. The filter coefficients (denoted as *c*_0_ to *c*_15_ in Figure 4C) are set in the register file and have a word width of 16 bits. Each FIR tap has an accumulator resolution of 26 bits (denoted as plus-sign in Figure 4C). The output of each FIR tap is sent to the FIR data wrap which itself is mapping the FIR tap output to the appropriate channel index. The detailed block diagram of one FIR tap is shown in Figure 4C.

#### 2.3.4 Adaptive threshold estimator

Figure 4D illustrates the block diagram of the Adaptive Threshold Estimator, comprising primarily a NEO module, a Zero Crossing (ZC) module, a Noise Estimator (NE) module, and a threshold calculator module.

The primary purpose of introducing the NEO module, as defined by Equation 6, is to enhance the detection of spike activity amidst background noise. Typically, spikes exhibit sudden changes in amplitude compared to noise.


(6) $$\begin{array}{l} \text{Ψ}\left[x\left(n\right)\right]=x{\left(n\right)}^{2}-x\left(n-1\right)\cdot x\left(n+1\right) \end{array}$$

where Ψ[*X*(*n*)] denotes the output of the NEO module corresponding to the neural signal *x*(*n*). As per Zeinolabedin et al. (2022) and Mukhopadhyay and Ray (1998), the NEO enhances the signal-to-noise ratio of the signal, thereby reducing its sensitivity to a threshold value, as observed in scenarios lacking NEO (Obeid and Wolf, 2004; Gibson et al., 2012; Zeinolabedin et al., 2015).

The Zero Crossing module serves to tally the zero crossings in the output of the NEO module, subsequently utilizing this count to estimate the firing rate. When consecutive points possess different sign bits, the counter within the Zero Crossing module increments by one. To more accurately represent the spike rate in hardware, a logarithmic operation is employed.

The Noise Estimator module primarily estimates the noise level present in the output of the NEO module. Initially, a low-pass filter within this module is applied to eliminate spikes, facilitating a more precise assessment of the noise level.

Subsequently, the threshold calculator module amalgamates the outcomes of the Zero Crossing and Noise Estimator modules to compute the corresponding threshold. This approach comprehensively accounts for the impact of spike rate and background noise on the threshold. By enhancing spike detection accuracy, it dynamically adjusts the thresholds of the analog frontend and spike detector modules, thereby optimizing the power consumption of the frontend acquisition.

The ATE is shared among 68 channels and operates independently for the single channel selected at any given time. Consequently, a working frequency of 20 kHz proves adequate, resulting in low power consumption of the ATE.

#### 2.3.5 Spike raster

The spike raster module is tasked with producing spike raster plots, which visualize the spiking patterns of a neural ensemble across time.

The block diagram of the SR system is depicted in Figure 4E. Upon initiating SR with the respective command, the Central Bio-Signal Processing Unit control logic continuously monitors the detection bits of each sample obtained from all 68 analog frontends, following the sequence from channel index 0–67. Subsequently, the observed results are relayed to the SR module. Within the SR module, an OR-gate operation is conducted across the 68 detection bits. If no spikes are detected in any channel, an empty packet with a designated packet header is transmitted to the Central Bio-Signal Processing Unit control logic. Conversely, in the event of spike detection, a packet indicating the firing channels along with timing details is dispatched to the Central Bio-Signal Processing Unit control logic. Within the Central Bio-Signal Processing Unit control logic, packets received from the SR are transmitted at an approximate frequency of 20 kHz. This implies that upon completion of a round of checking across all 68 analog frontends by the Central Bio-Signal Processing Unit control logic, a packet is constructed and conveyed via SPI.

### 2.4 MAC-assisted processing element

#### 2.4.1 Processing element

**Figure 5:**
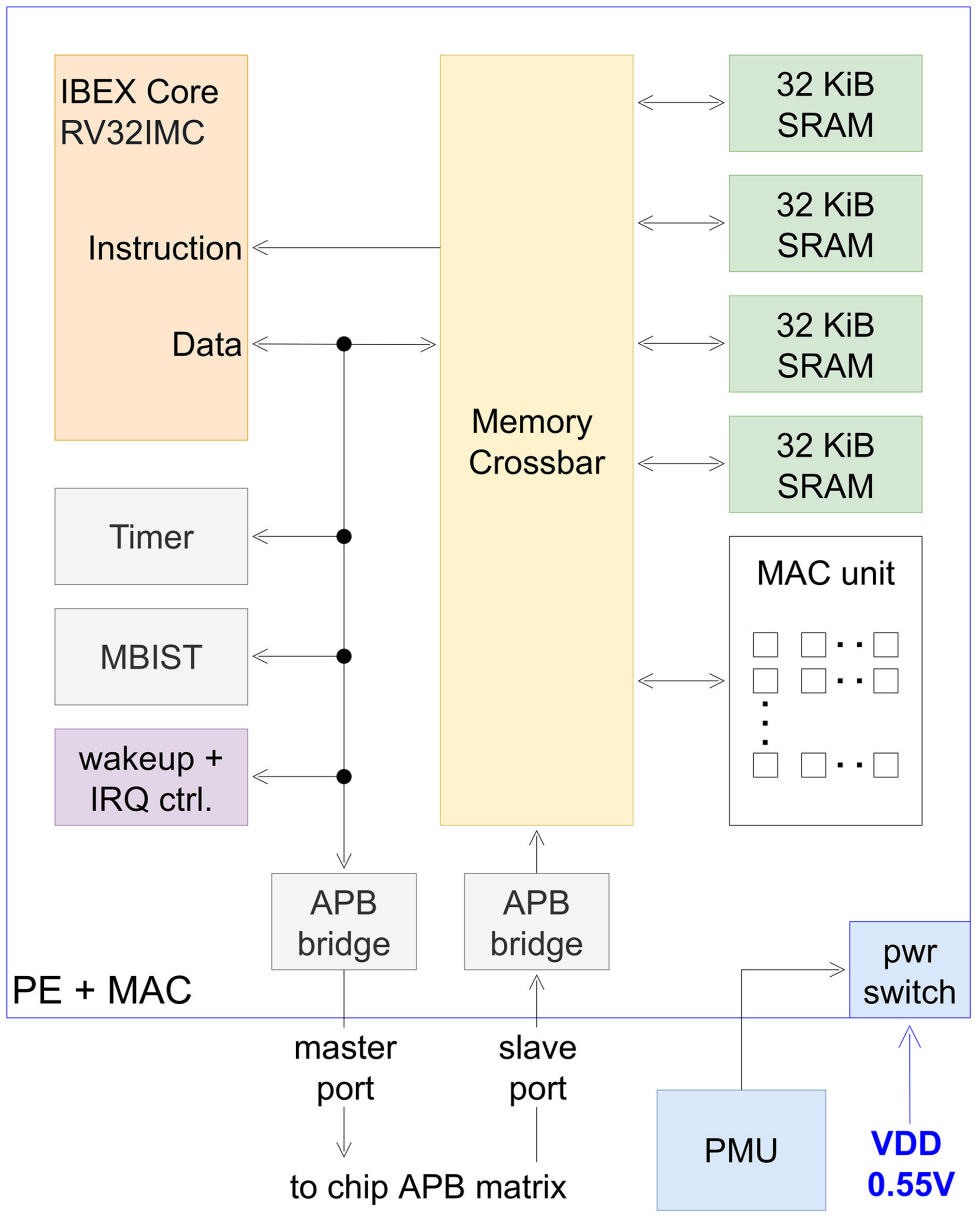
Block diagram of processing element and MAC. IRQ, interrupt request; MBIST, memory built-in self test; PMU, power management unit.

Our RISC-V PE architecture is shown in Figure 5 and was adapted from Bauer et al. (2023). The main design and implementation goal was to reduce static- as well as dynamic- power consumption. For this purpose, an adaptive reverse body biasing scheme (Walter et al., 2020) was utilized at 0.55 V supply voltage (VDD). Moreover, power switches are implemented, to power-gate the PE if not needed. The used IBEX RISC-V core supports RV32IMC instructions and achieves an architectural performance of 3.12 CoreMarks/MHz. To reduce leakage power, the PE is implemented with a target frequency of 25 MHz using only regular-Vt standard cells. A 128 KiB SRAM organized in four banks of 32 KiB SRAM can be used flexibly as instruction and data memory. The SRAM can be accessed by the PE and MAC, as well as other chip components via the APB bus, allowing various data flow scenarios. To test SRAM memory after production, a memory built-in self test block was implemented. A flexible wake-up and interrupt controller allows to clock-gate internal components while the PE is sleeping. To further reduce static power consumption, a retention sleep mode was implemented. This allows to power down SRAM periphery while retaining memory contents. Various interrupt sources (timer, MAC, APB access and external interrupt request lines) can be used to wake up the PE from its sleep modes.

#### 2.4.2 MAC unit

**Figure 6:**
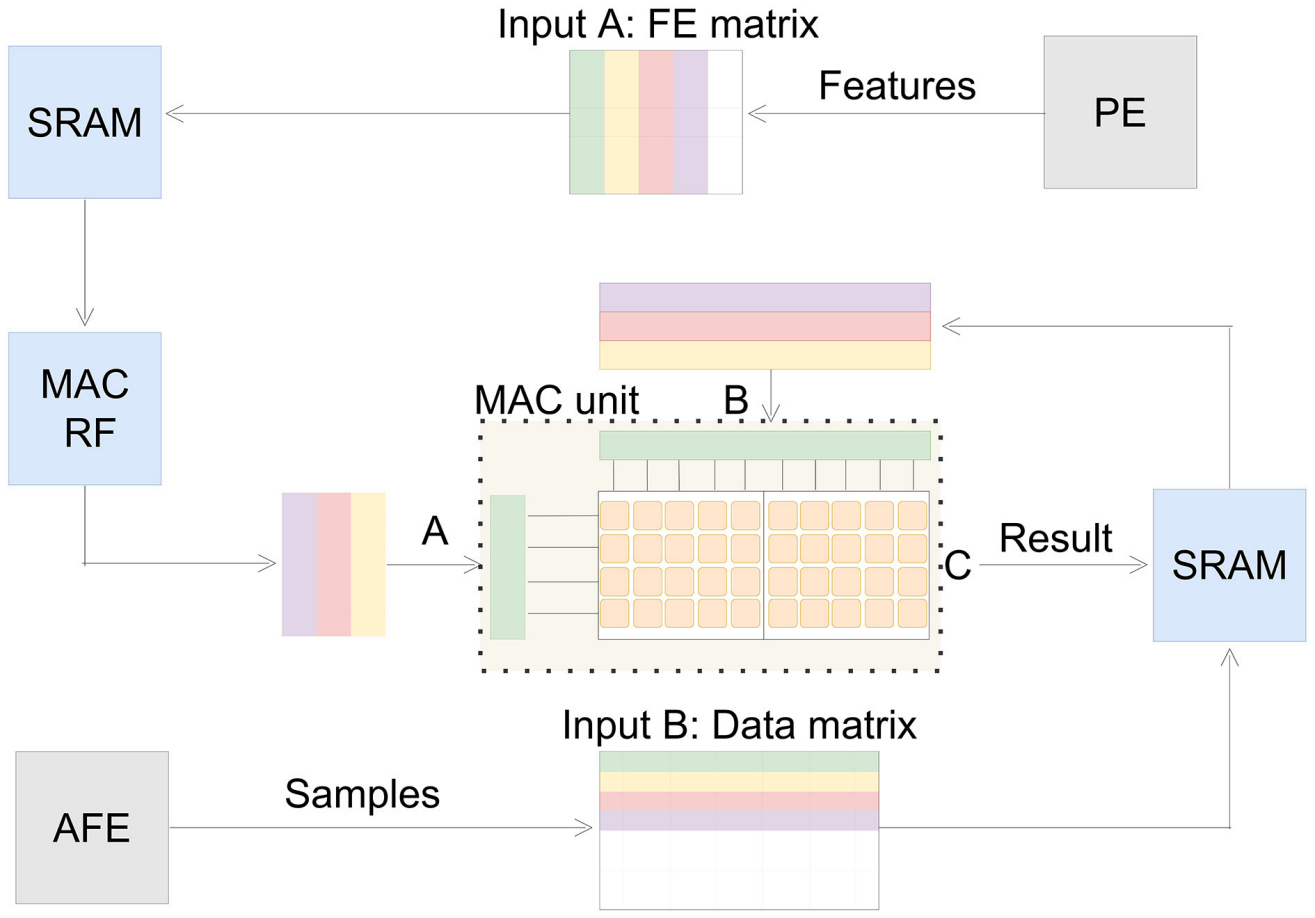
Dataflow of the MAC unit. The feature extraction matrix is stored in SRAM or the internal register file depending on the working flow. Upon being triggered by the PE, the MAC unit executes the required multiply-accumulate operations in the specified order (from green to purple) and then writes the results (C) back into the SRAM.

The MAC unit is designed to support various neural signal processing applications. Though the MAC unit can work independently, it functions as a support module for the RISC-V. Therefore it requests and sends data from SRAM as shown in Figure 6. To scale down memory requests, the MAC unit is caching its feature matrix from SRAM into internal register files, as presented in Figure 6. This reduces time and power consuming memory requests in inference mode, when utilized for spike sorting. Because only samples are requested from SRAM in this case. To ensure high accuracy for the processing the accumulator of the MAC unit has a bit size of 32. The MAC unit consists of multiple cells processing 9x16-bit signed operations in parallel. To increase energy efficiency the PE can be set into sleep mode during the execution of the MAC unit. The control, input- and output-dimensions and features of the MAC unit are accessible through registers. This way the whole processing chain of neural signal processing is efficiently controlled and synchronized with each other.

#### 2.4.3 Spike sorting

**Figure 7:**
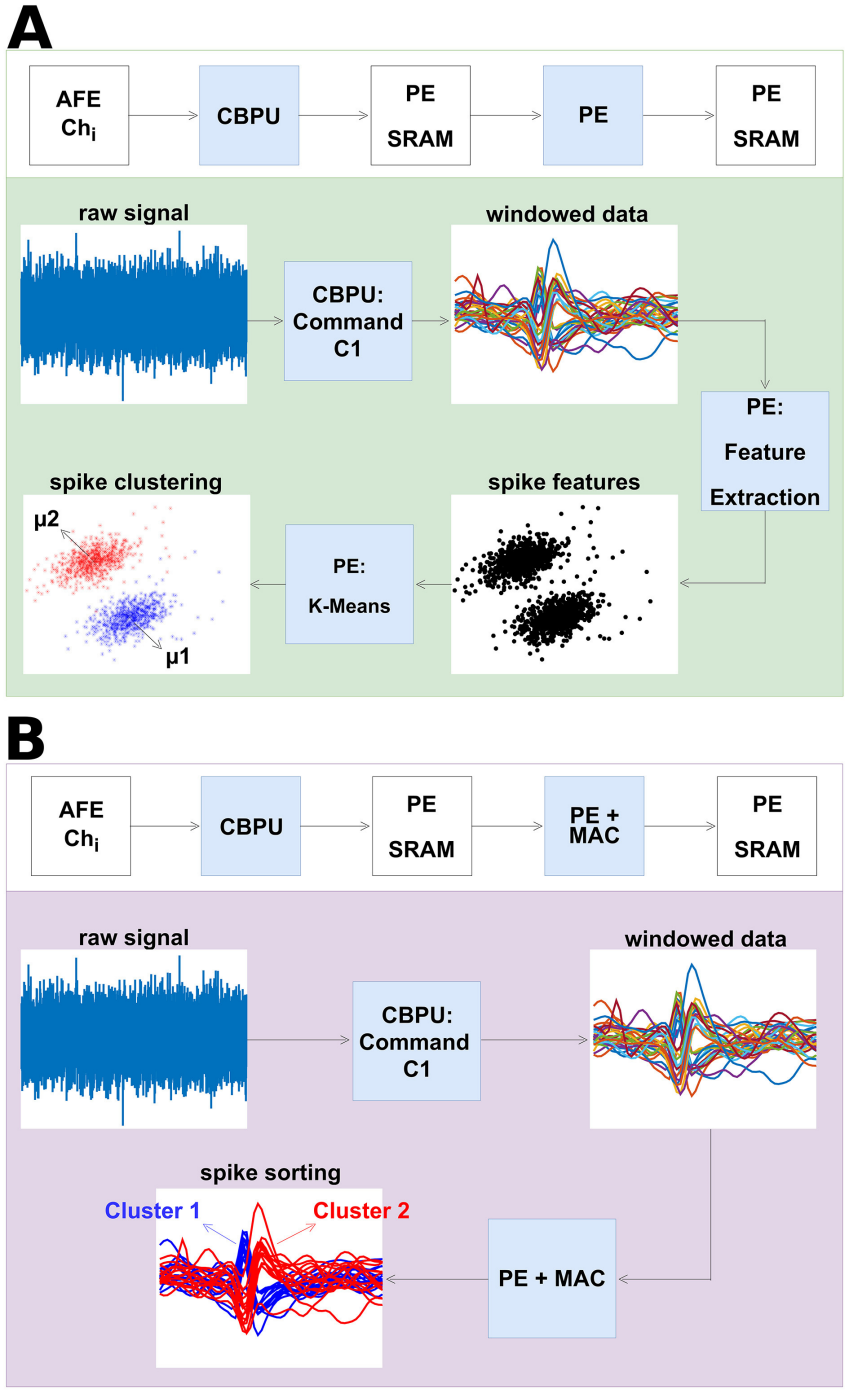
Illustration and process of spike sorting. **(A)** The training process of the neural spike sorting in PE flow. The neural signals are recorded from analog frontends and stored in SRAM memory. In this instance, the PE computes spike features via PCA and partitions the occurring spikes into clusters via K-means clustering. **(B)** The inference process of the neural spike sorting in PE and MAC flow. The usage of the MAC unit improves the energy efficiency of spike sorting by around 23.8% compared to performing these spike sorting steps in PE alone.

As introduced, spike sorting plays a crucial role in processing action potentials, the training and inference flows of which are shown in Figure 7. The MAC unit here serves to accelerate the spike sorting algorithm, supporting various feature extraction methods such as principal component analysis (PCA) and adaptive filter (AF), offering hence increased flexibility for on-chip spike sorting. It receives data from SRAM memory and can be deployed in different feature extraction methods, in which multiply-accumulate operations are the basis. For instance, in this work, PCA in combination with improved K-means described in Do et al. (2019) was selected to demonstrate feature extraction in the neural spike sorting and clustering algorithm, as depicted in Figure 7A. During the training mode, the principal components of the neural signal are stored in SRAM. Figure 7B illustrates the inference flow of spike sorting. PCA is sensitive to outliers. To handle this issue, the processing element either normalizes the raw data prior to PCA or applies post-PCA techniques such as density-based spatial clustering of applications with noise (DBSCAN; Liu et al., 2024). In this process, the MAC unit conducts feature extraction on spikes utilizing the training results, aiding in the spike sorting procedure. The distance calculation in the spike sorting process is carried out by the PE.

**Figure 8:**
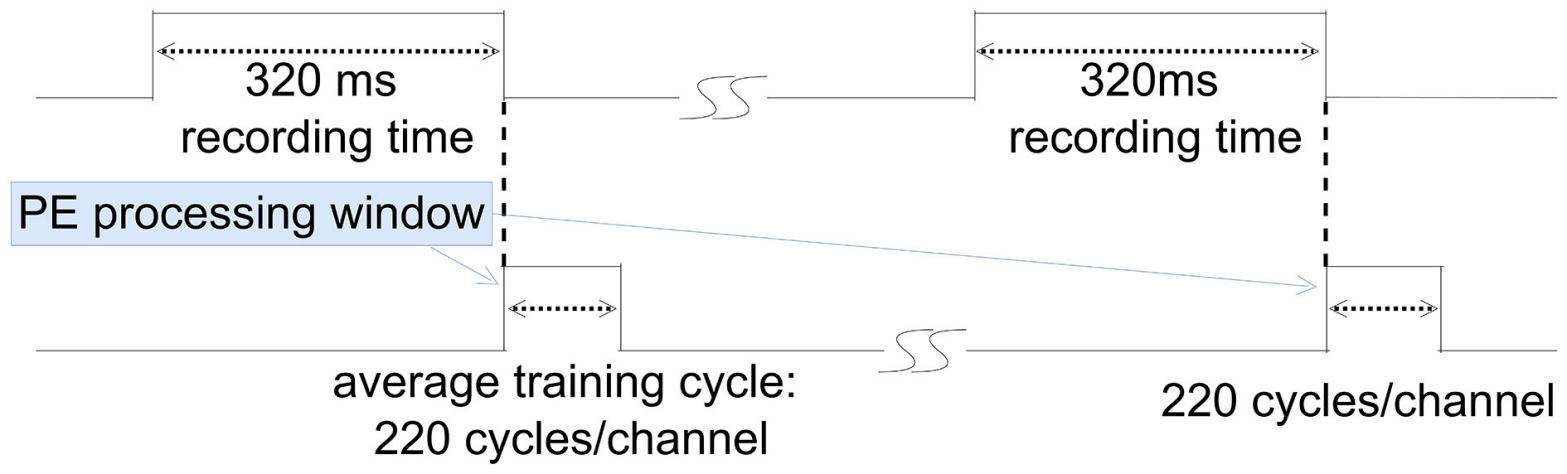
Training time analysis.

PCA proves to be an efficient feature extraction method for atypical datasets, streamlining feature selection during the training process (Glaser and Marks, 1968; Abeles and Goldstein, 1977). However, in most scenarios, AF emerges as a more efficient and straightforward feature extraction method (Zeinolabedin et al., 2022). Therefore, AF is generally chosen as the feature extraction method in our work. Using AF, spikes are aligned at the maximum slope points. It takes an average of 220 clock cycles per channel to process an input sample, as illustrated in Figure 8. During the recording phase and MAC operating phase, the PE remains in sleep mode. In inference mode, the MAC unit computes the projection of recorded spike samples into the spike feature space. During this phase, only sampled spikes are transmitted to SRAM, fetched by the MAC unit, and entered onto the predefined and already cached feature extraction matrix.

The advantage of software-based spike sorting extends beyond flexibility in feature extraction to include the inference process as well. While most spike sorting hardware engines solely support Euclidean distance metric, which may be insufficient for certain datasets (Guo et al., 2022), the software-based spike sorting in this study can accommodate complex distance metrics such as Mahalanobis distance metric. The corresponding distance (Euclidean or Mahalanobis) of spikes to each cluster mean in the feature domain is calculated and the minimum distance determines the spike sorting result.

In our experiments, as noted by Pedreira et al. (2012), the number of clusters is capped at a maximum of 8. The number of features can be set up to 10 for PCA and up to 7 for AF (Guo et al., 2022; Zeinolabedin et al., 2016). For most noisy datasets, Mahalanobis distance metric outperforms the Euclidean distance metric by ~10%, though with increased computational complexity (Guo et al., 2022).

### 2.5 Recording frontends

**Figure 9:**
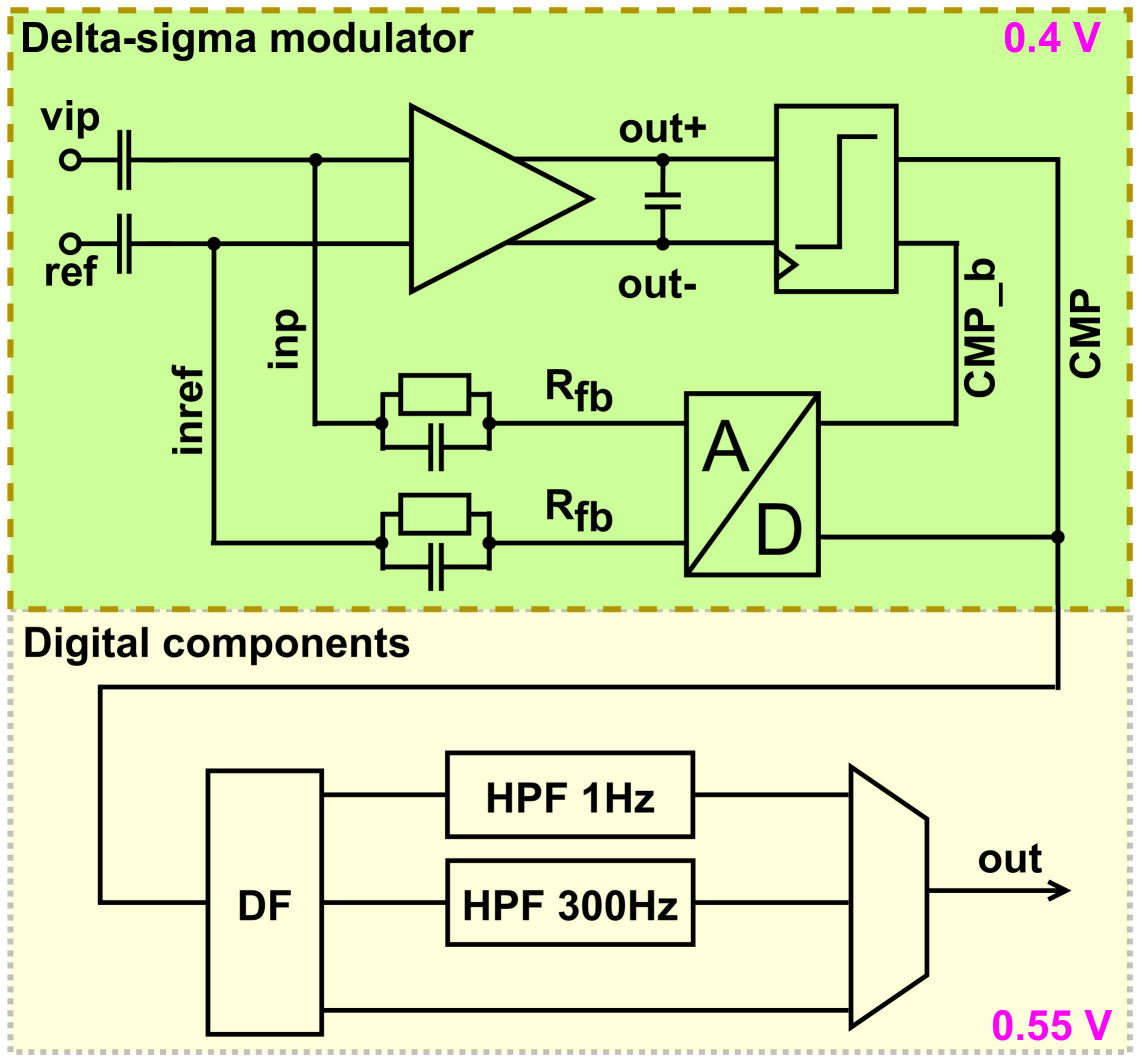
Architecture of analog frontend.

Figure 9 shows the architecture of the recording frontend. Action potentials and/or LFP are sampled through 68 continuous-time delta-sigma ADCs with corresponding digital high- and low-pass filter acting as decimation filters. The latter can be set to high bandwidth- (10 kHz) or low bandwidth-mode (2.4 kHz). The delta-sigma modulator runs at 0.4 V, by applying forward biasing at the back-gates (Schüffny et al., 2023b). Its input range, derived from capacitive voltage dividers, spans from -1 to 1 mV, resulting in a least significant bit of 3.9 μV. Its current and sampling frequency are reduced in low-bandwidth mode. The feedback is a parallel resistor-capacitor network to form a high-pass filter with the input decoupling capacitors. The resistor is implemented as a pseudo resistor, which defines the DC voltages at the input of the integrator. The output of the integrator is digitized with a 1 bit comparator. Thus the digital-to-analog converter to close the loop has the same resolution. The sampling frequency in low-bandwidth mode is 1.25 and 5 MHz in high-bandwidth mode. Since low-bandwidth mode is sufficient to record spikes, it is used in this paper. The implemented high-pass filter can be set to 300 Hz to remove local field potential, 1 Hz to remove offset only or be disabled for characterization purposes. Implementing the filters digitally, increases PVT robustness significantly, in comparison to analog filter implementations. To save power, the first stage of the decimation filter is a cascaded integrator-comb filter with an asynchronous implemented integrator. The second stage is a polyphase FIR filter with a decimation of two followed by another polyphase FIR filter with a decimation of two. The asynchronous integrator as well as the polyphase approach reduce switching and thus power dynamic current in the circuit. The digital components are supplied by 0.55 V. The delta-sigma modulators are combined with a digital-on-top approach combined with the digital filters to the ADC macro. This macro is instantiated 68 times on toplevel enabling further scaling for more channels. Details about a similar analog frontend are presented in Schüffny et al. (2022, 2023b).

Regardless of the recording previous publications, show the capability of implementing neural stimulators in 22 nm FDSOI (Schüffny et al., 2023a), enabling a closed-loop system on a chip.

## 3 Results

**Figure 10:**
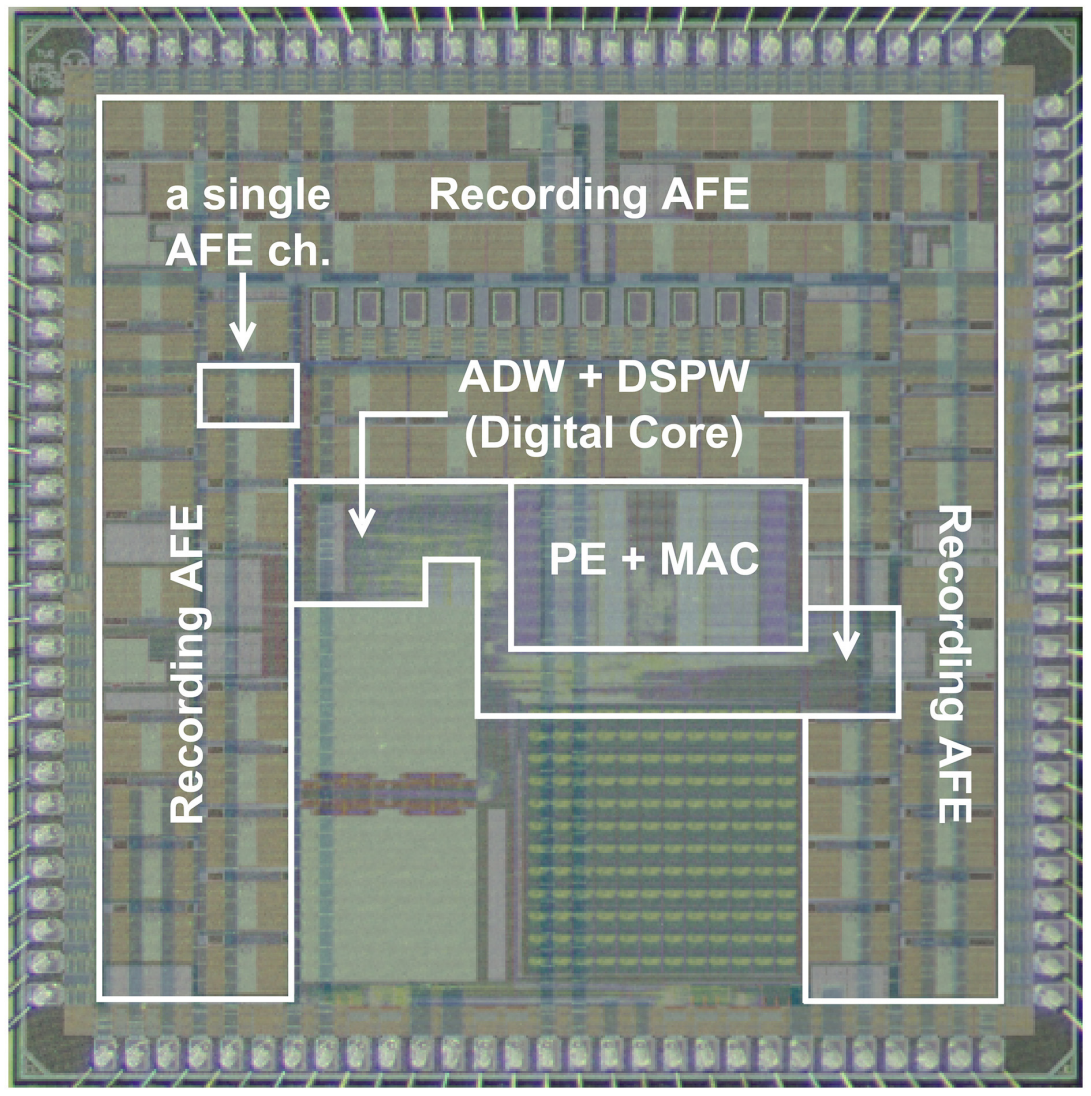
Chip photo, 3 mm × 3 mm, components marked, and transparent layout overlaid.

The proposed neural signal recording and processing system has been implemented using 22 nm GlobalFoundries FDSOI technology. Figure 10 displays the chip photo, annotated with components and overlaid with the transparent layout. This section presents power and area measurements, as well as a comparison with other relevant works.

### 3.1 Setup for measurement and validation

**Figure 11:**
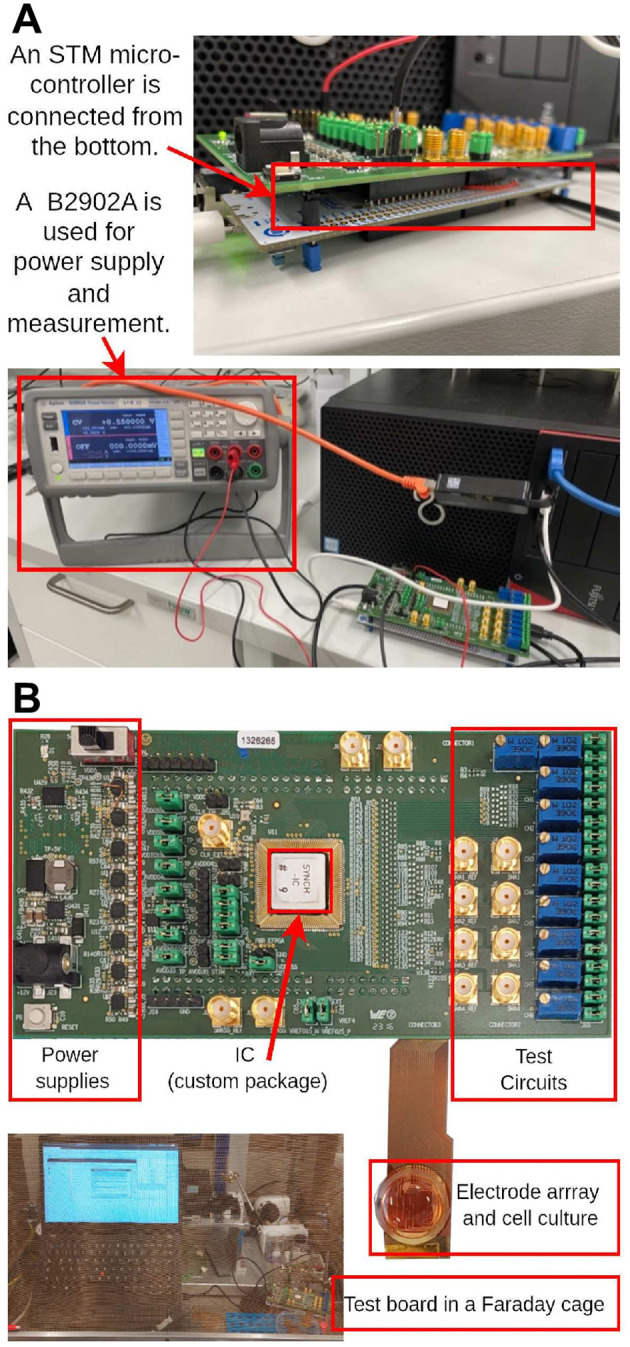
Setup for chip test, measurement and *in vitro* experiments. **(A)** Setup for chip power measurement. **(B)** Top view of the chip along with PCB board, electrode array and cell culture, accompanied by a photograph depicting the recording environment utilizing our chip.

The different operating modes and functional units were tested as depicted in Figure 11A. An STM microcontroller is connected at the bottom of the printed circuit board (PCB) board, serving as a bridge for data communication between the board and a computer. During testing, datasets are initially read and converted to analog signals in the testbench to serve as inputs. To evaluate the power consumption, a B2902A precision lab power supply was used to supply the 0.55 V domain. All measurements were carried out at room temperature of 20°C.

The chip is connected to an electrode array and cell culture to enable the actual recording of neuron activities *in vitro*. To safeguard against external electromagnetic interference, a Faraday cage is employed to shield the chip, its associated electronics, and the electrodes, as shown in Figure 11B.

**Figure 12:**
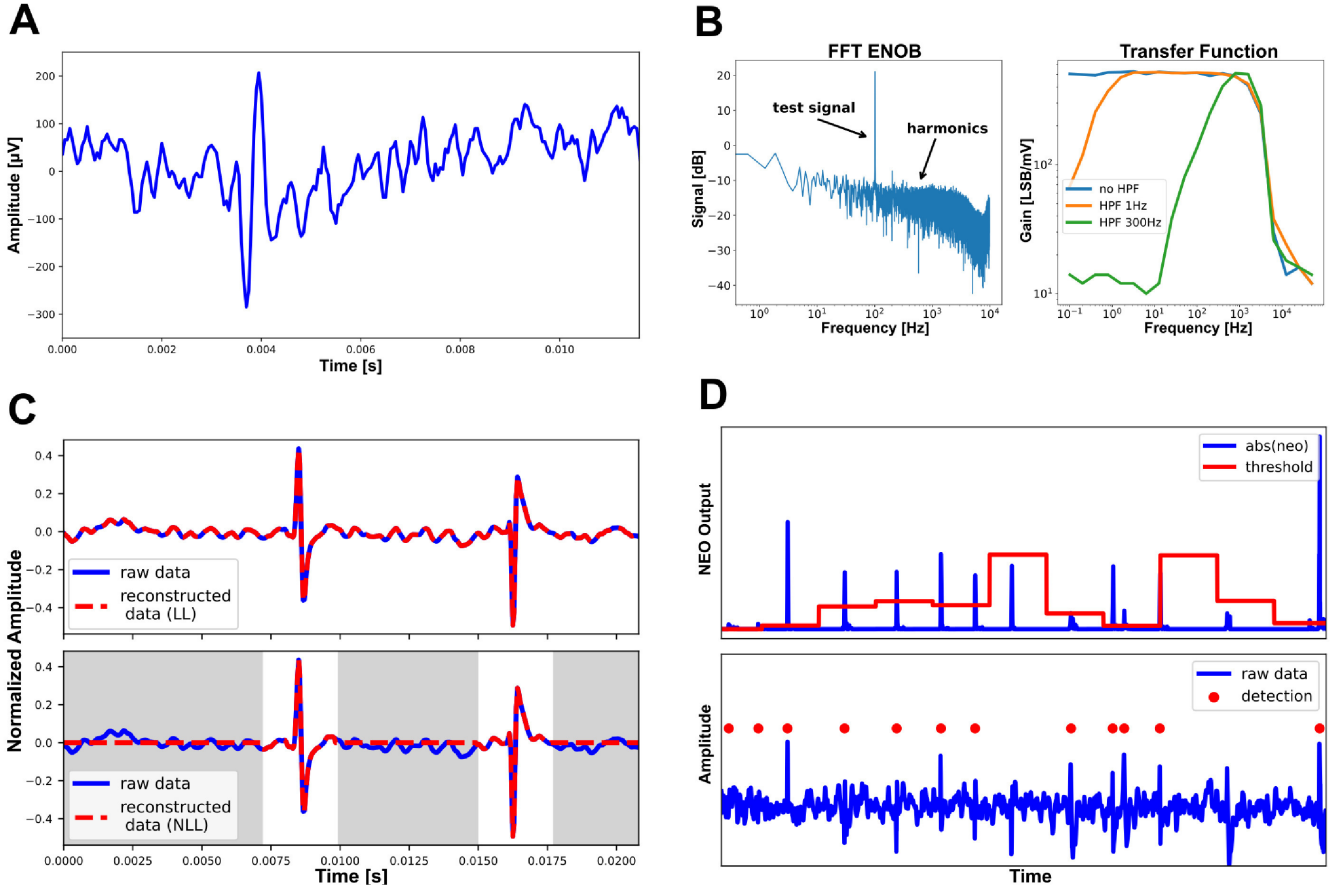
Experimental results. **(A)** Recording of an artificial spike, replayed through a Tektronix AFG3252 waveform generator. **(B)** Characterization of the analog frontend. **(C)** Losless and near-lossless compression. **(D)** Adaptive threshold estimation and spike detection. ENOB, effective number of bits; FFT, fast Fourier transform; LL, lossless; NLL, near-lossless.

Figure 12 presents experimental results. Figure 12A showcases the recording of an artificial Spike, replayed through a Tektronix AFG3252 waveform generator. The differential signal was provided through a 1:100 resistive divider, to bring it into the analog frontends voltage range, and applied to a recording channel and its corresponding reference. Figure 12B characterizes the analog frontend. Figure 12C illustrates the near-lossless compression mode. The gray-labeled parts represent non-spiking parts and are treated as zeros. Figure 12D demonstrates the results of the adaptive threshold estimation and spike detection. The threshold is dynamically estimated every 64 samples (configurable) and represented by the red line in the upper part of the subfigure. Once updated, the threshold is stored in the AFEs register file. A spike is detected when the absolute output of the NEO module exceeds this threshold. The lower part of the subfigure shows the corresponding data segment (blue line) and the detected spikes (red points).

### 3.2 Power and area

As emphasized, the efficiency in power consumption and area of programmable SoC holds paramount importance in assessing the practicality and viability of systems within the neuroprosthetics domain. Hence, we undertake a comprehensive characterization of the digital domain of the proposed programmable SoC with regard to power and area in this subsection. The power consumption of all digital processing units is measured at 0.55 V supply voltage by applying the ABB technique.

The Intra-Channel Compression Engine comprises two distinct compression engines utilizing AC and GC techniques, respectively. Generally, AC requires a maximum of 120 clock cycles to compress a single symbol, whereas GC requires significantly fewer clock cycles. Given an acquisition frequency of 20 kHz, Intra-Channel Compression Engine necessitates a minimum frequency of 2.4 MHz. However, the near-lossless mode imposes a more stringent timing constraint. As elucidated in Section 2.3, in the worst-case scenario of the near-lossless mode, 64 samples must be compressed within a recording time of 32 samples. Hence, to ensure real-time processing of neural signals, a frequency of 5 MHz is required. Under this condition, each AC exhibits an average dynamic power of 3.50 μW/Ch coupled with a leakage power of 0.89 μW/Ch in the lossless mode. In the near-lossless mode, the dynamic power is reduced to 3.24 μW/Ch. In contrast, the GC engine demonstrates greater power efficiency, albeit with a slight drop in SSR. In the lossless mode, each channel incurs an average dynamic power of 0.54 μW/Ch and a leakage power of 0.33 μW/Ch, while in the near-lossless mode, the dynamic power is measured at 0.42 μW/Ch. For Cross-Channel Compression Engine, despite the fewer clock cycles required by GC, a single GC engine is shared among 8 channels. Hence, a frequency of 5 MHz is maintained. The 8-channel Cross-Channel Compression Engine consumes a dynamic power of 1.86 μW and a leakage power of 0.70 μW, totaling 0.32 μW/Ch. The FIR filter operates across 16 channels in parallel. To meet real-time processing demands, a frequency of 50 MHz is selected. The average total power consumption of the 16-channel FIR filter is measured at 25.76 μ W.

**Figure 13:**
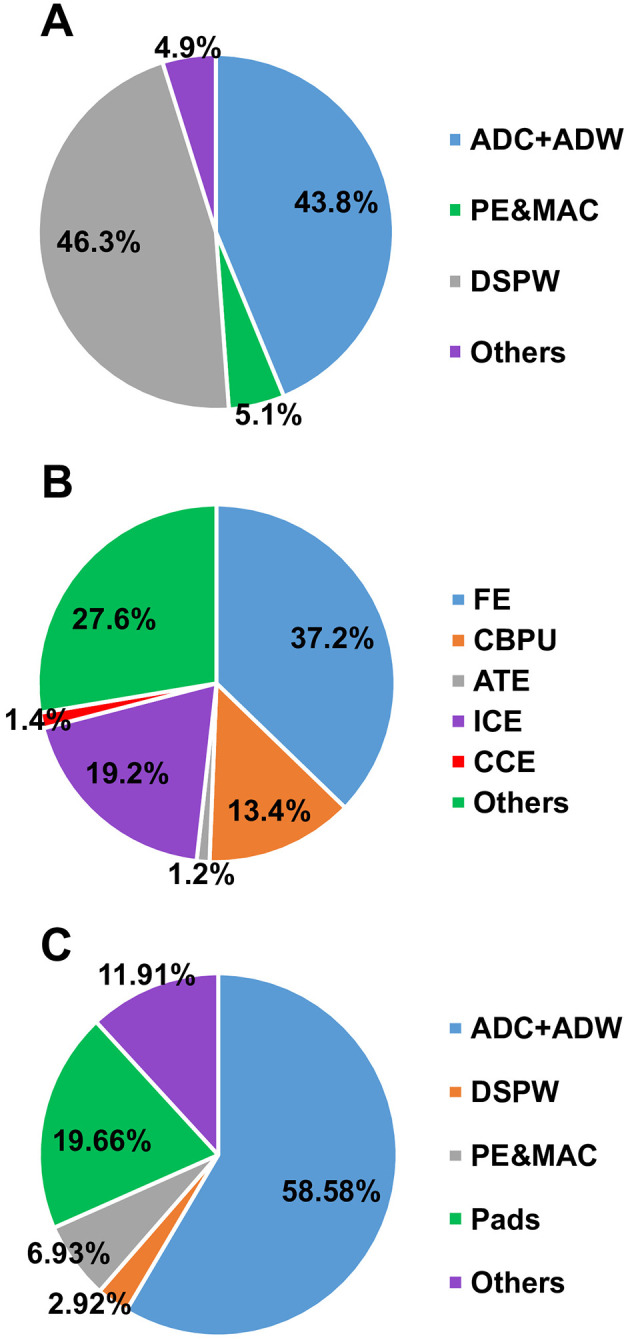
Leakage power and area break-down to characterize the programmable SoC. **(A)** The leakage power break-down of the proposed programmable SoC. **(B)** The leakage power break-down of the Digital Signal Processing Wrapper block. **(C)** The chip area break-down.

The leakage power break-down of the proposed programmable SoC is shown in Figure 13A and the leakage power of the Digital Signal Processing Wrapper is shown in Figure 13B.

**Figure 14:**
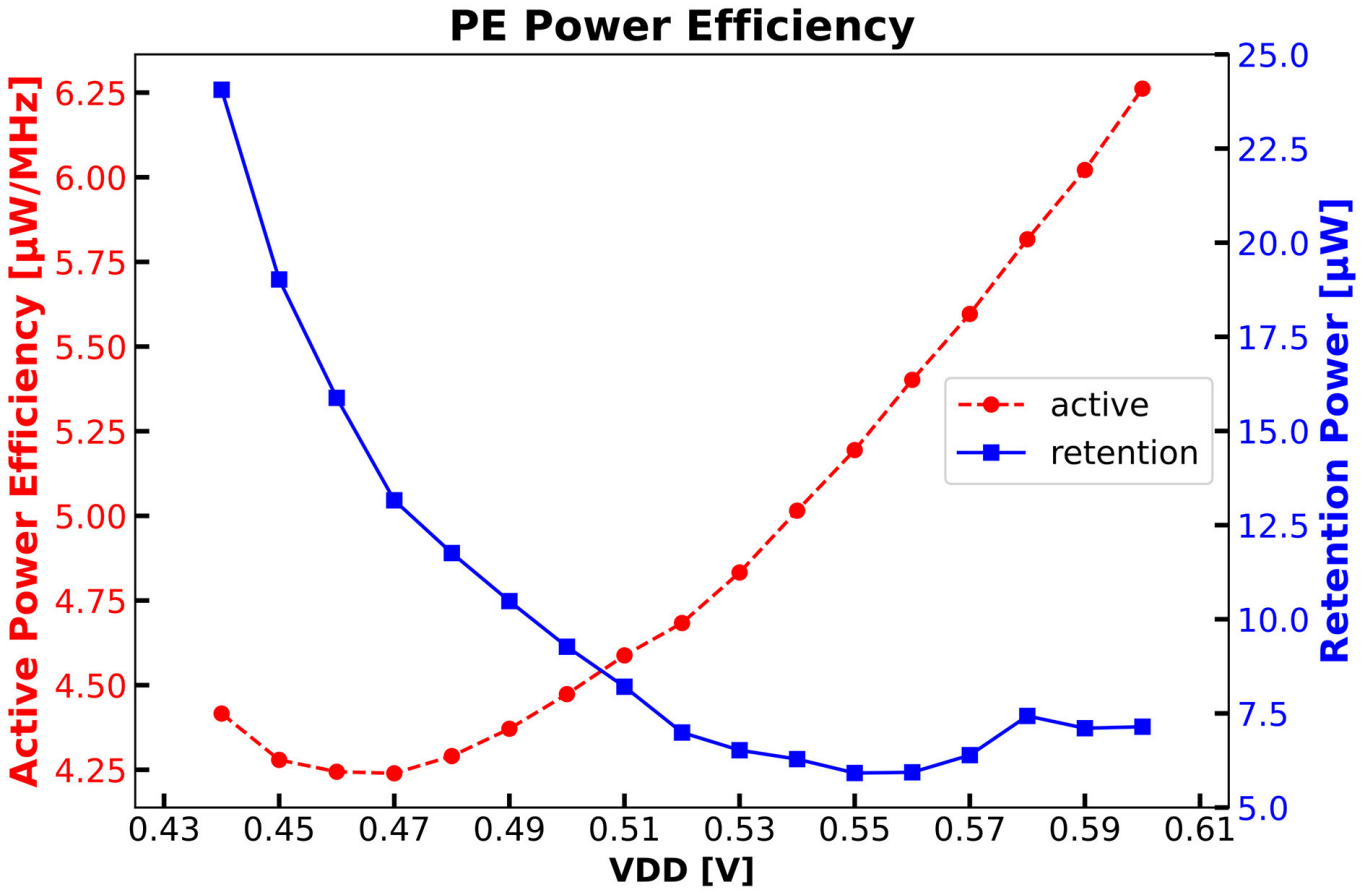
PE power efficiency running CoreMark^@^ 25 MHz and PE sleep power during retention sleep mode.

Figure 14 shows the PE power efficiency running CoreMark at various VDD voltages. We achieve 5.19 μW/MHz at a nominal VDD of 0.55 V. By reducing VDD to 0.47 V the efficiency is 4.24 μW/MHz. As shown in Figure 14 the retention sleep power consumption has its minimum of 5.91 μW at nominal VDD. A reduction of VDD causes the ABB regulation to adapt the back bias voltage to meet speed requirements resulting in higher cell leakage. As previously introduced, the PE offers significant on-chip programmability, facilitating tasks such as training for Cross-Channel Compression Engine and both training and inference for spike sorting. The on-chip training of Cross-Channel Compression Engine consumes 16.94 μJ. Meanwhile, the on-chip training of a software-based spike sorting process requires 2.4 nJ/sample. Similarly, the inference process incurs an energy consumption of 1.43 μJ/spike. With the utilization of the MAC accelerator, energy consumption is enhanced by 23.8% to 1.09 μJ/spike.

The chip area break-down is shown in Figure 13C. It depicts that the analog frontend plus Analog-Digital Wrapper dominates the area. Conversely, the area occupied by the Digital Signal Processing Wrapper is minimal in comparison. Consequently, the proposed programmable SoC imposes a modest area overhead when juxtaposed with traditional neural implants. This observation underscores the scalability and adaptability of the proposed on-chip processing engines.

## 4 Discussion

### 4.1 Comparison

A comparison with other state-of-the-art neural signal acquisition and processing chips is presented in Table 1. From the comparison, it is evident that the proposed programmable SoC exhibits a greater integration of on-chip neural processing approaches. Additionally, for each individual approach, our programmable SoC offers comparable or superior results.

**Table 1:** Comparison with state-of-the-art designs.

**Works**	**This**	**A-SSCC 2022**	**JSSC 2022**	**TBioCAS 2022**	**ISSCC 2023**	**JSSC 2022**
	**work**	**(Schüffny et al., 2022)**	**(Uran et al., 2022)**	**(Zeinolabedin et al., 2022)**	**(Chen et al., 2023)**	**(Shin et al., 2022)**
Tech. (nm)	22	22	65	22	22	65
Volt. (V)	AFE: 0.4,	AFE: 0.4,	1.0	AFE: 0.8,	AFE: -,	AFE: 1.2,
	Dig.: 0.55	Dig.: 0.55		Dig.: 0.5-0.8	Dig.: 0.59	Dig.: -
Number of AFE ch.	68	64	16	16	No	256
Compression	AP & LFP	AP	AP & LFP	No	No^†^	No
Spike detection	2-stage (Adap. Thr. & NEO)	2-stage (Adap. Thr. & NEO)	Hard thresholding	NEO	NEO	No
Feature extraction	PCA/AF/SW-based	No	CHT	AF	Peak-FSDE	HT/BPF
Spike sorting	SW-based	No	No	Adap. SC	Geo-OSort	No
ATE area (mm^2^)	0.0031	0.0058	No	No	No	No
CE-SSR	AP: 63% (LL)/	AP: 62.5% (LL)/	AP/LFP:	No	No^†^	No
	91% (NLL)^§^,	91% (NLL),	80%			
	LFP: 64% (LL)	LFP: No	(Lossy)			
CE-Power/Ch.	AP: 0.87 - 4.39,	AP: 16.47 - 17.84,	AP & LFP:	No	No^†^	No
(μW)	LFP: 0.32	LFP: No	1.83			
CE-Area/Ch.	AP: 0.0026/0.0064,	AP: 0.147,	AP & LFP:	No	No^†^	No
(mm^2^)	LFP: 0.00043	LFP: No	0.0076			
PE-Energy (CE-Training, μJ)	16.94	No	No	No	No	No
PE-Energy (SS^*^ -Training, μJ)	15.36	No	No	28.46	No	No
SS-Accuracy	91.48%/94.12% ^‡^	No	92%/97.8% ^¶^	94.12%	89.5%	95.6%
SS-Datasets	Quiroga	No	CHB-MIT	Quiroga	Quiroga	CHB-MIT
	(Quiroga et al., 2004)		(Shoeb and Guttag, 2010)			
AFE-Power (μW)	0.41	0.40	0.65	1.52	No	1.51

^†^The spike sorting is categorized as a form of compression in this paper. However, it is only the index of spiking neurons and is not reconstructive.

^§^For datasets with low noise levels, AC achieves an SSR of up to 74.5% in LL mode and 93.9% in NLL mode, while GC achieves an SSR of up to 69.5% in LL mode and 93.2% in NLL mode, respectively.

^*^SS, spike sorting.

^‡^Two distinct feature extraction algorithms are employed here.

^¶^The accuracy here was performed off-chip.

In the proposed programmable SoC, 68 recording frontends are integrated, surpassing those in Schüffny et al. (2023b), Zeinolabedin et al. (2022), and Uran et al. (2022), thereby enabling the recording of a larger number of neural signal channels. Furthermore, the power consumption of 0.41 μW, encompassing both the analog frontend and digital wrapper, also demonstrates advantages compared to almost all state-of-the-art alternatives.

The on-chip Digital Signal Processing Wrapper in this work demonstrates remarkable versatility and efficiency. For action potential compression, our programmable SoC offers two modes: The lossless mode and the near-lossless mode. Compared to the lossless mode, where both spikes and non-spiking segments can be fully reconstructed, the near-lossless mode treats the non-spiking parts as zeros, allowing only the waveform and timing information of spikes to be accurately reconstructed. In comparison to Uran et al. (2022), our approach achieves a higher SSR of 91% in the near-lossless mode, while strictly preserving spike waveforms. Additionally, compared to Schüffny et al. (2023b), although the SSR is similar, our compression engine exhibits significant advantages in terms of area and power consumption per channel, making it highly applicable. For LFP compression, despite having a lower SSR than Uran et al. (2022), our programmable SoC holds three distinctive features: (1) significantly smaller area, (2) substantially lower power consumption, and (3) preservation of signal reconstruction integrity with no loss. Our spike detector operates in two stages, offering greater stability compared to the hard thresholding method employed in Uran et al. (2022), as well as the single NEO stage documented in Zeinolabedin et al. (2022) and Chen et al. (2023). When compared to the 2-stage spike detector used in Schüffny et al. (2023b), our Adaptive Threshold Estimator occupies only half the area. Furthermore, the spike sorting capability of our programmable SoC achieves a satisfactory accuracy of 91.48 or 94.12% over the Quiroga datasets in Quiroga et al. (2004), which is comparable to the accuracies achieved by other methods using the same datasets. Additionally, our programmable SoC includes an efficient FIR filter and spike raster, expanding its range of potential applications.

Ultimately, the integrated ultra-low power MAC-assisted PE offers programmability and versatility for a multitude of applications, including on-chip training for Cross-Channel Compression Engine, as well as on-chip training and inference for spike sorting. Compared to Zeinolabedin et al. (2022), our programmable SoC achieves lower energy consumption for tasks such as spike sorting.

### 4.2 Conclusion

In this paper, we introduce a highly-integrated neural signal processing programmable SoC fabricated using 22nm FDSOI technology, occupying a compact chip size of 9 mm^2^. Leveraging advanced ABB technology, our design achieves remarkable ultra-low power consumption. The architecture of our programmable SoC encompasses 68-channel analog frontends, a digital processing wrapper, and a MAC-assisted PE. The digital processing wrapper features a robust command-based central control unit and functional units, facilitating comprehensive on-chip processing of neural signals with exceptional energy efficiency. This includes tasks such as lossless/near-lossless compression of AP, lossless compression of LFP, filtering of raw neural signals for LFP extraction, generation of spike raster plots, and adaptive estimation of spike detection thresholds to enhance the performance of the spike detection module. Notably, the analog frontend consumes a mere 0.25 μW/Ch, while the Analog-Digital Wrapper consumes only 0.16 μW, housing digital filters, spike detectors, and FIFO buffers. The proposed programmable SoC achieves an SSR of ~91% for action potentials in the near-lossless mode and an SSR of 64% for LFP losslessly, consuming only 0.87 (GC)–4.39 (AC) μW/Ch and 0.32 μW/Ch, respectively. The 16-channel FIR filter exhibits an average power consumption of 25.76 μW. Furthermore, the on-chip MAC-assisted PE offers programmability and versatility for various applications, including on-chip training for Intra-Channel Compression Engine and Cross-Channel Compression Engine, as well as on-chip training and inference for spike sorting. The on-chip training for the Cross-Channel Compression Engine module consumes an average of 16.94 μJ. The on-chip training of a software-based spike sorting requires 2.4 nJ/sample. Correspondingly, the inference process consumes 1.43 μJ/spike. By employing the bio-specific MAC accelerator, the energy consumption is improved by 23.8% to 1.09 μJ/spike, achieving an average accuracy rate of 91.48 or 94.12% based on the utilized features. The PE consumes a power of 5.19 μW/MHz at a supply voltage of 0.55 V. By reducing the supply voltage to 0.47 V, the efficiency is 4.24 μW/MHz. The retention sleep mode has a power consumption of ~5.91 μW. Compared to conventional neural implants, the programmable SoC proposed in this work significantly reduces data rate and transmission power consumption, while offering diverse on-chip processing capabilities, thereby enabling the potential increase in the number of recording channels for a new class of cortical active and intelligent implants.

Future work centered around the created signal recording- and analysis-platform involves the real-time classification of neuronal firing dynamics under different environments for drug screening applications *in vitro*, using the setup shown in Figure 11.

## Data Availability

Publicly available datasets were analyzed in this study. This data can be found at: https://figshare.le.ac.uk/articles/dataset/Simulated_dataset/11897595; https://buzsakilab.nyumc.org/datasets/.
