# Supplementary material for: 68-channel neural signal processing system-on-chip with integrated feature extraction, compression, and hardware accelerators for neuroprosthetics in 22 nm FDSOI
Source: Front Neurosci. 2024 Oct 23;18:1432750. doi: 10.3389/fnins.2024.1432750 (PMC11541109; doi:10.3389/fnins.2024.1432750)
Supplement: Supplementary file 1 [file Data_Sheet_1.pdf]

Supplementary Material

LIST OF ABBREVIATIONS

ABB	Adaptive Body-Biasing
AC	Arithmetic Coding
ADC	Analog-to-Digital Converter
ADW	Analog-Digital Wrapper
AF	Adaptive Filter
AFE	Analog Frontend
AHB	Advanced High-Performance Bus
AP	Action Potential
APB	Advanced Peripheral Bus
CBPU	Central Bio-Signal Processing Unit
CCE	Cross-Channel Compression Engine
CE	Compression Engine
DB	Data Buffer
DF	Digital Filter
DPCM	Differential Pulse-Code Modulation
DPCM2	2nd-order Differential Pulse-Code Modulation
DSA	Delta-Sigma Analog-to-Digital Converter
DSPW	Digital Signal Processing Wrapper
ENOB	Effective Number of Bits
FDSOI	Fully Depleted Silicon-On-Insulator
FE	Feature Extraction
FFT	Fast Fourier Transform
FIR	Finite Impulse Response
FIFO	First-In-First-Out
GC	Golomb Coding
GPIO	General Purpose Input/Output
HPF	High-Pass Filter
ICE	Intra-Channel Compression Engine
IRQ	Interrupt Request
KiB	Kibibyte
LFP	Local Field Potential
LL	Lossless
LPF	Low-Pass Filter
MAC	Multiply-Accumulate
MBIST	Memory Built-In Self Test
Mbps	Megabits per Second
MCDE	Multi-Channel Decorrelation Engine
MCU	Microcontroller Unit
MUA	Multi-Unit Activity
MUX	Multiplexer

NE	Noise Estimator
NEO	Nonlinear Energy Operator
NLL	Near-lossless
PCA	Principal Component Analysis
PCB	Printed Circuit Board
PMU	Power Management Unit
PVT	Process Voltage and Temperature
RF	Register File
RISC-V	Reduced Instruction Set Computer
RLE	Run-Length Encoding
SD	Spike Detector
SoC	System-on-Chip
SPI	Serial Peripheral Interface
SR	Spike Raster
SRAM	Static Random Access Memory
SS	Spike Sorting
SSR	Space Saving Ratio
VDD	Supply Voltage
ZC	Zero Crossing
